# Supplementary material for: Postprandial FGF19-induced phosphorylation by Src is critical for FXR function in bile acid homeostasis
Source: Nat Commun. 2018 Jul 3;9:2590. doi: 10.1038/s41467-018-04697-5 (PMC6030054; doi:10.1038/s41467-018-04697-5)
Supplement: Supplementary file 1 — Supplementary Information [file 41467_2018_4697_MOESM1_ESM.pdf]

Supplementary information

Byun et al. Postprandial FGF19 signaling-induced FXR phosphorylation by Src is critical for its bile acid homeostatic function

Supplementary Figure 1

a

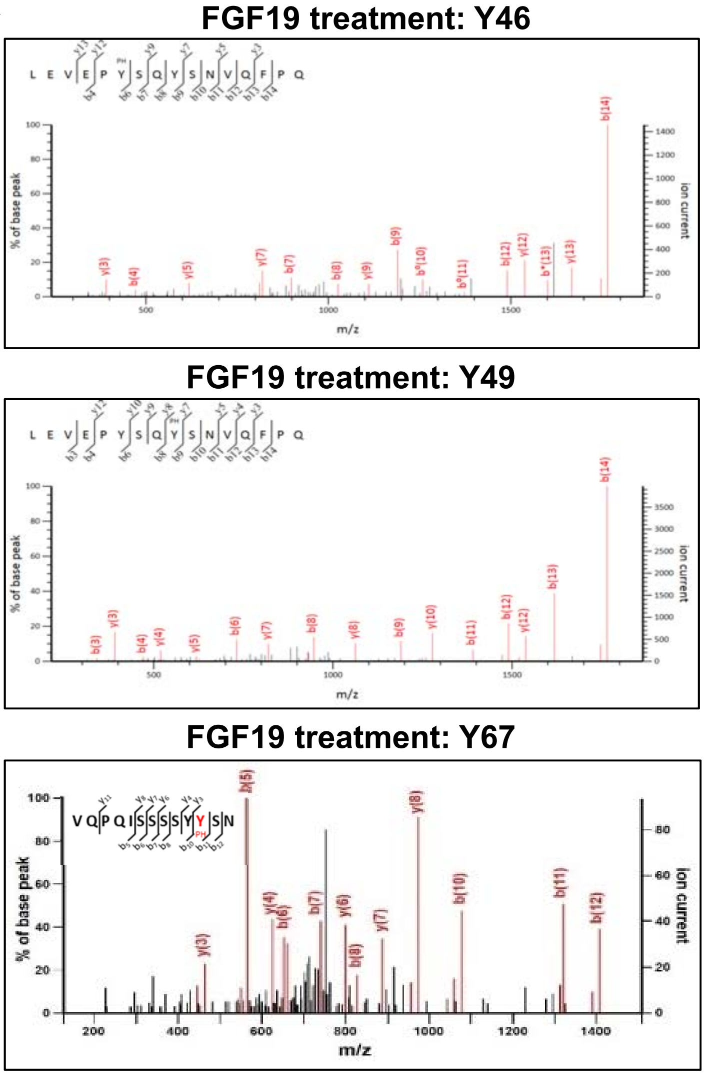

b

Tyrosine Site Phosphorylation Prediction

Position	Sequence	Score
46	EVEPYSQYS	0.694
49	PYSQYSNVQ	0.643
66	SSSSYYSNL	0.893
**67**	**SSSYYSNLG**	**0.968**
73	NLGFYPQQP	0.028
81	PEEWYSPGI	0.438
86	SPGIYELRR	0.320
97	AETLYQGET	0.727
137	RASGYHYNA	0.830
139	SGYHYNALT	0.114
161	KNAVYKCKN	0.634
174	VMDMYMRRK	0.606
198	AECMYTGLL	0.196
264	IMDSYNKQR	0.284
365	ISDEYITPM	0.836
373	MFSFYKSIG	0.397
386	TQEEYALLT	0.673
401	PDRQYIKDR	0.222

c

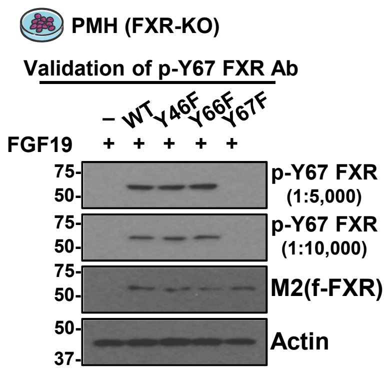

d

FXR Seqeunces

**Human**	54	FPQVQPQISSSSY**Y**SNLGFYPQQPEEW	80
**Mouse**	64	FPQVQPQISSSSY**Y**SNLGFYPQQPEDW	90
**Rat**	50	FPQVQPQISSSSY**Y**SNLGFYPQQPEDW	76
**Monkey**	64	FPQVQPQISSSSY**Y**SNLGFYPQQPEEW	90
**Dog**	63	FPQVQPQISSSSY**Y**SNLGFYPQHPEEW	89
**Cow**	64	FPQVQPQISSSSY**Y**SNVGFYPQQPEEW	90
**Chicken**	55	FPQVQPQISSPPY**Y**SNLGFYPPQHEEW	81
**Zebrafish**	59	YTSGEPSMSSPSY**Y**SSQHCYSQYGAEE	85

Supplementary Figure 1. FXR is phosphorylated at Tyr-67 in response to FGF19 treatment. (a) Flag-human FXR was adenovirally expressed in primary mouse hepatocytes (PMH) isolated from FXR-KO mice and 48 h later, the cells were treated with 5 μM MG132 for 4 h to inhibit proteasomal degradation of FXR and then treated with 50 ng ml^{-1} FGF19 for 10 min. Flag-FXR was purified using M2 agarose (Sigma, Inc) and subjected to proteomic analysis. FXR peptides modified by phosphorylation that were identified by LC-MS/MS analysis. Phosphorylated Y46 (LEVEPYSQYSNVQFPQ), Y49 (LEVEPYSQYSNVQFPQ) and Y67 (VQPQISSSSYYSN) were detected in samples from cells treated with FGF19. **(b)** Predicted tyrosine phosphorylation sites in FXR were identified using NetPhos 3.1. The most highly predicted site at Y67 is in bold. **(c)** Validation of the p-Y67 FXR specific antibody: Flag-FXR WT and phosphorylation defective mutants were expressed in FXR-KO mouse PMH. Cells were treated with 50 ng ml^{-1} FGF19 for 10 min, and proteins were detected with the p-Y67-specific antibody by IB. **(d)** Comparison of amino acid sequences adjacent to Y67 in the indicated vertebrate species.

Supplementary Figure 2

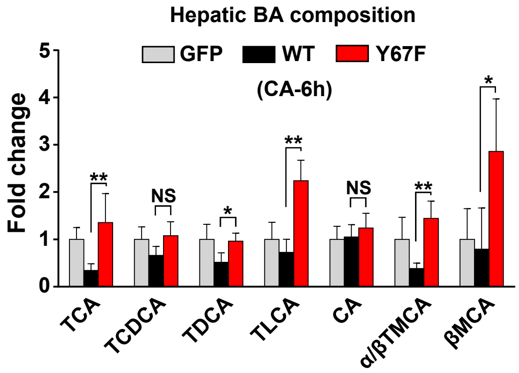

Supplementary Figure 2. Effects of the phosphorylation-defective Y67F mutation of FXR on hepatic BA profiles. GFP, FXR WT or Y67F-FXR was expressed in FXR floxed mice infected with AAV-TBG-Cre and the mice were feed CA-containing chow for 6 h. Levels of BAs in the liver were determined by metabolomic analysis. TCA, taurocholic acid; TCDCA, taurochenodeoxycholic acid; TDCA, taurodeoxycholic acid; TLCA, taurolithocholic acid; CA, cholic acid; α/βTMCA, α/β-tauromuricholic acid; βMCA, β-muricholic acid. Values are presented as mean ± SD. Statistical significance was measured using one-way ANOVA with the Bonferroni post-test. *P<0.05, **P<0.01, and NS, statistically not significant (n= 5).

Supplementary Figure 3

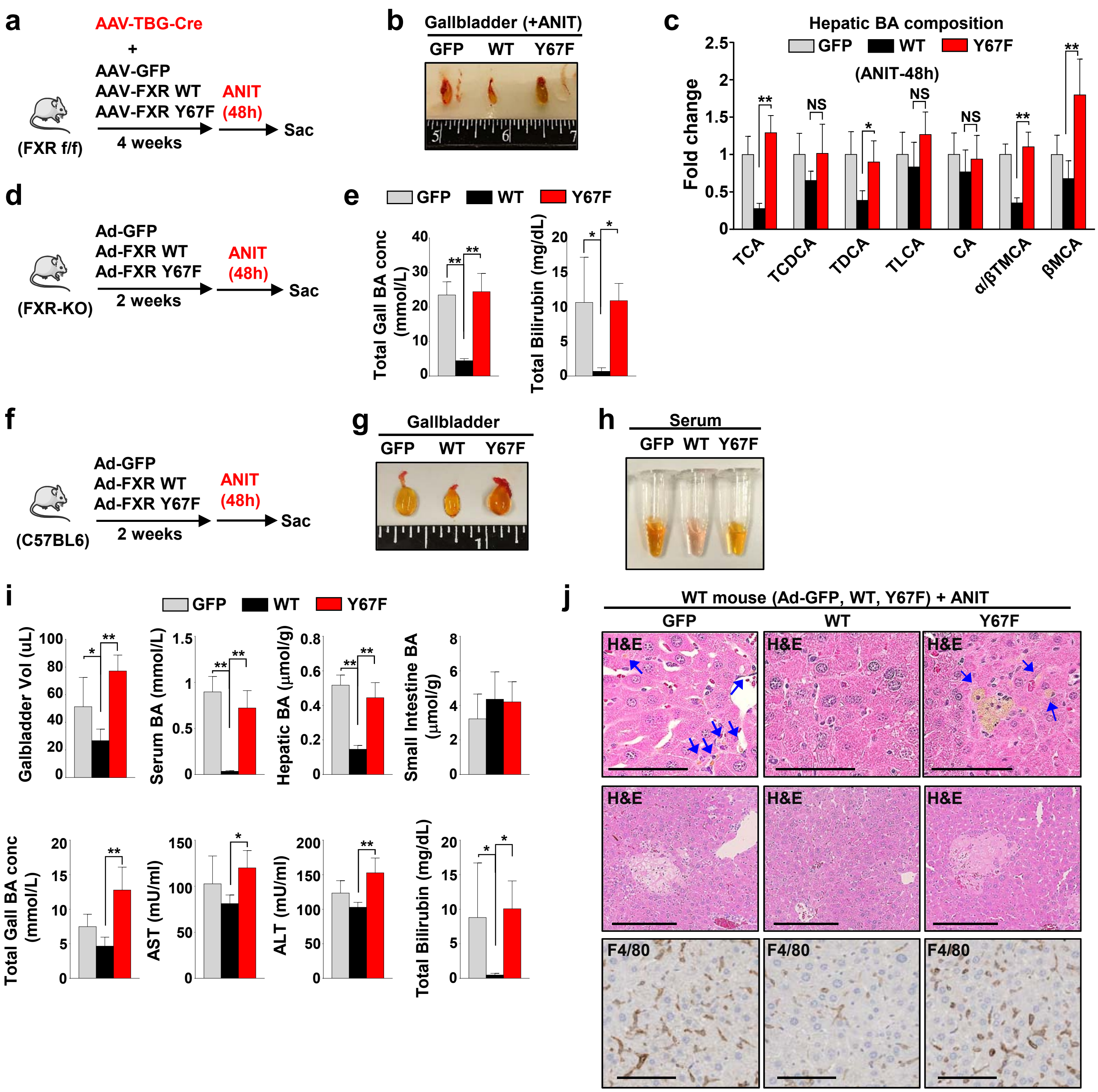

Supplementary Figure 3. The phosphorylation-defective Y67F mutation of FXR exacerbates cholestatic symptoms upon biliary insult. (a) Experimental outline. **(b)** Image of gallbladder from mice. **(c)** Total hepatic bile acid (BA) composition from FXR floxed mice infected with AAV-TBG-Cre and expressing GFP, FXR WT or Y67F-FXR after 48 h of ANIT treatment. TCA, taurocholic acid; TCDCA, taurochenodeoxycholic acid; TDCA, taurodeoxycholic acid; TLCA, taurolithocholic acid; CA, cholic acid; α/βTMCA, α/β-tauromuricholic acid; βMCA, β-muricholic acid. **(d)** Experimental outline. **(e)** Total gallbladder BA levels and total bilirubin levels were determined (n= 6 mice). **(f)** Experimental outline. **(g, h)** Images of gallbladders and serum from ANIT-treated C57BL/6 mice expressing GFP, FXR WT or Y67F-FXR. **(i)** The volume of gallbladders, serum, hepatic, and small intestine BA levels, total gallbladder BA levels and serum aspartate transaminase (AST), alanine transaminase (ALT) and total bilirubin levels (n= 6 mice). **(j)** Liver sections were stained with haematoxylin and eosin (H&E) to detect necrosis, and macrophages were detected (brown) with F4/80 antibody. Abnormally accumulated bile is indicated by the arrows. Scale bar, 80 µm (top), 200 µm (middle) and 100 µm (F4/80). All values are presented as mean ± SD. **(c,e,i)** Statistical significance was measured using one-way ANOVA with the Bonferroni post-test. *P<0.05, **P<0.01, and NS, statistically not significant.

Supplementary Figure 4

a

Position in query protein	Sequence in query protein	Corresponding motif described in the literature (phosphorylated residues in red)	Features of motif described in the literature	Link to original article describing the motif
1	44 - 46	EPY	[E/D]XpY	SHP1 phosphatase substrate motif
2	46 - 47	YS	pY[A/G/S/T/E/D]	Src kinase substrate motif
3	49 - 50	YS	pY[A/G/S/T/E/D]	Src kinase substrate motif
4	49 - 52	YSNV	pYXX[L/I/V]	JAK2 kinase substrate motif
5	49 - 54	YSNVQF	pYXXXX[F/Y]	ALK kinase substrate motif
6	65 - 70	SYYSNL	[I/V/L/S]XpYXX[L/I]	**Src family kinase substrate motif**
7	66 - 67	YY	[E/D/Y]pY	TC-PTP phosphatase substrate motif
8	67 - 68	YS	pY[A/G/S/T/E/D]	**Src kinase substrate motif**
9	67 - 70	YSNL	pYXX[L/I/V]	JAK2 kinase substrate motif
10	67 - 72	YSNLGF	pYXXXX[F/Y]	ALK kinase substrate motif
11	78 - 81	EEWY	[E/D]XXpY	ALK kinase substrate motif
12	79 - 81	EWY	[E/D]XpY	SHP1 phosphatase substrate motif
13	81 - 82	YS	pY[A/G/S/T/E/D]	Src kinase substrate motif
14	81 - 86	YSPGIY	pYXXXX[F/Y]	ALK kinase substrate motif
15	86 - 87	YE	pY[A/G/S/T/E/D]	Src kinase substrate motif

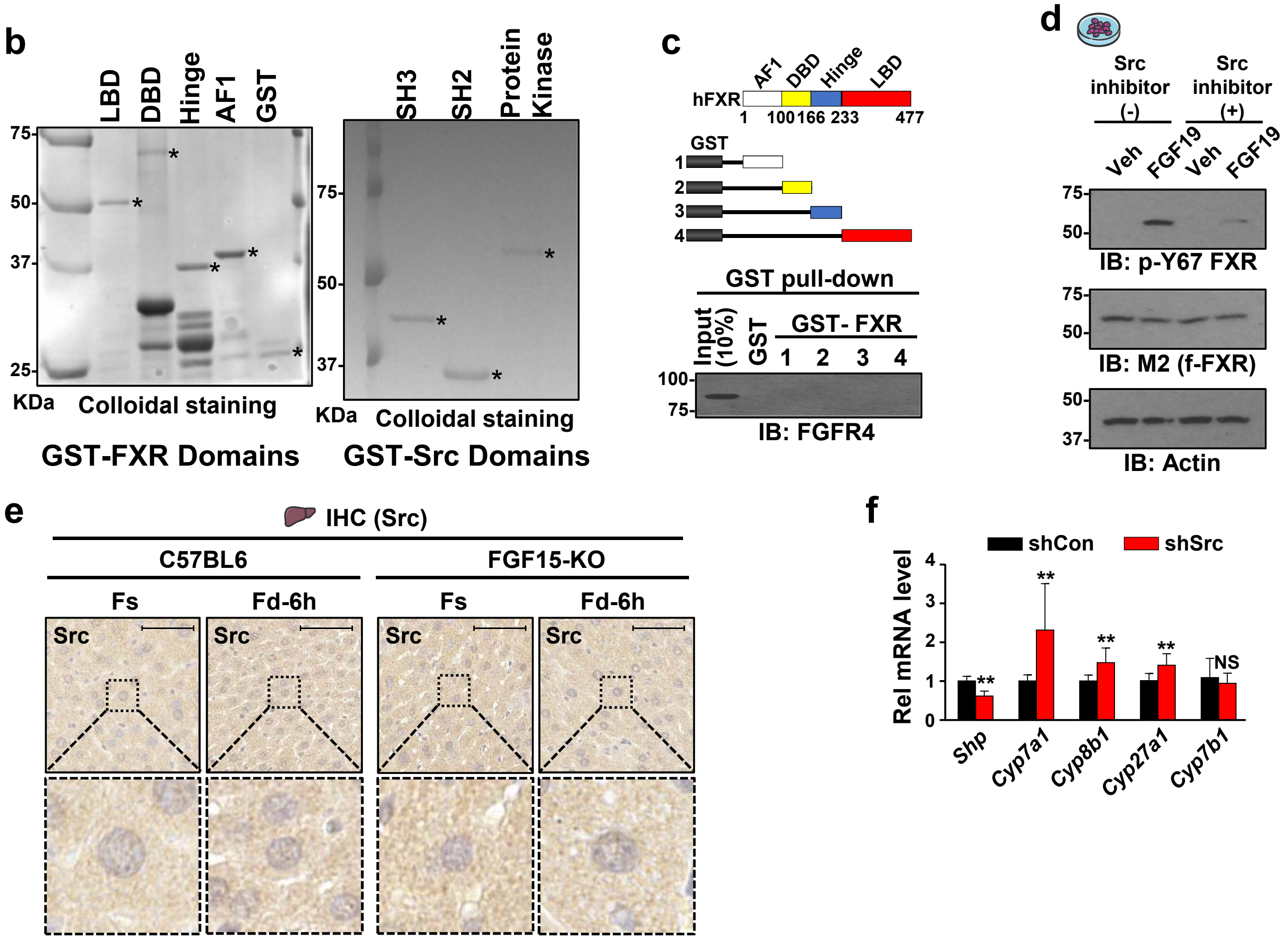

Supplementary Figure 4. Role of c-Src kinase in FXR phosphorylation at Y67 in response to FGF19 treatment. (a) Prediction of possible phosphorylation sites and relevant kinases using the PhosphoMotif Finder program of the Human Protein Reference Database program (http://www.hprd.org). **(b)** GST-FXR and GST-Src fusion proteins used in GST pull down interaction studies were visualized by Colloidal Blue staining. LBD, ligand-binding domain; DBD, DNA-binding domain. **(c)** Fragments of FXR were fused to GST (top). FGFR4 protein was synthesized in vitro using TNT system (Promega, Inc) and binding of FGFR4 to GST-FXR fusion proteins was detected by immunoblotting (IB). **(d)** PMH expressing flag-FXR were pre-treated with vehicle or 1 nM of dasatinib for 1 h and then treated with 50 ng ml^{-1} FGF19 for 10 min. The p-Y67 FXR levels were determined by IB. **(e)** Liver sections from fasted or fed C57BL/6 or FGF15-KO mice were analyzed by immunohistochemistry (IHC) using the Src antibody. Representative images are shown. Scale bar, 50 µm. **(f)** qRT-PCR: Lenti-shRNA for Src was expressed in mice by tail vein injection for 2 weeks and the mRNA levels of indicated genes in liver were measured by qRT-PCR (n= 5 mice). Statistical significance was measured using the **(f)** Mann-Whitney test. **P<0.01, and NS, statistically not significant.

Supplementary Figure 5

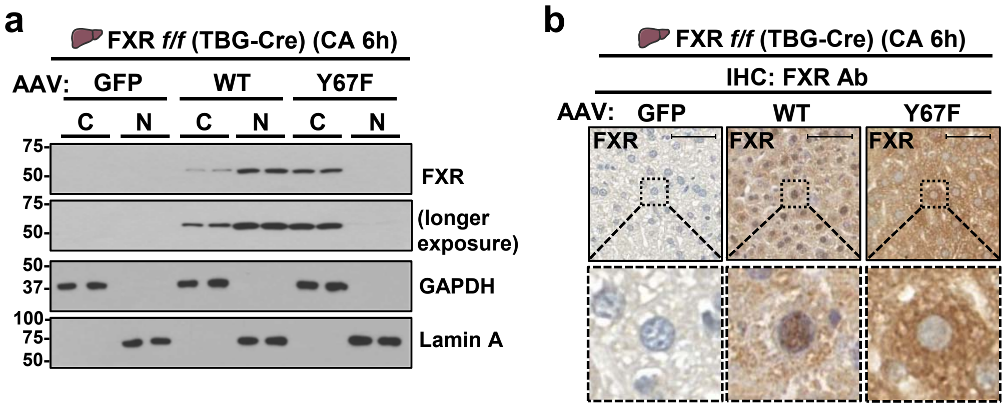

Supplementary Figure 5. Phosphorylation of FXR at Y67 is important for CA-induced nuclear localization of FXR. (a, b) FXR-WT or Y67F-FXR was expressed in FXR-floxed mice infected with AAV-TBG-Cre as described in Fig. 2a. **(a)** Nuclear (N) and cytoplasmic (C) extracts were isolated and levels of FXR in N or C fractions were measured by IB. **(b)** FXR in liver sections was detected by IHC. Scale bar, 50 μm.

Supplementary Figure 6

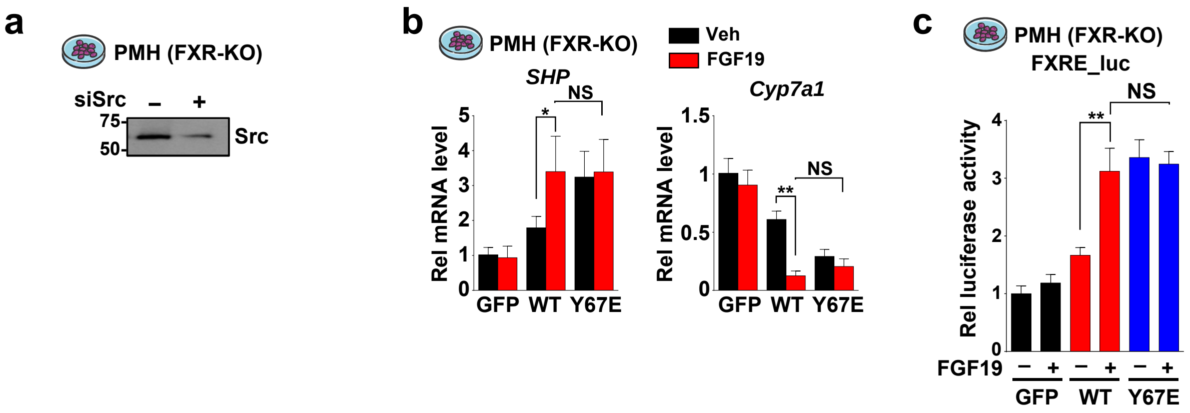

Supplementary Figure 6. Effects of the phosphorylation-mimic Y67E mutation on FXR transactivation. (a) PMH from FXR-KO mice were transfected with siRNA for Src as indicated and infected with Ad-flag-FXR, and then the cells were treated with 50 ng ml^{-1} FGF19 for 30 min. Src levels in input samples were detected by IB. **(b)** qRT-PCR: GFP, flag-FXR or the Y67E mutant were expressed with plasmids as indicated in FXR-KO PMH and then, cells were treated with vehicle or 50 ng ml^{-1} FGF19 for 30 min. The mRNA levels of indicated genes were measured by qRT-PCR (n= 5). **(c)** Luciferase reporter assay: PMH from FXR-KO mice were transfected with plasmids as indicated and then, cells were treated with vehicle or 50 ng ml^{-1} FGF19. Luciferase activities were determined and normalized to β-galactosidase activity (n= 4). All values are presented as mean ± SD. **(b, c)** Statistical significance was measured using two-way ANOVA with the Bonferroni post-test. *P<0.05, **P<0.01, and NS, statistically not significant.

Supplementary Figure 7

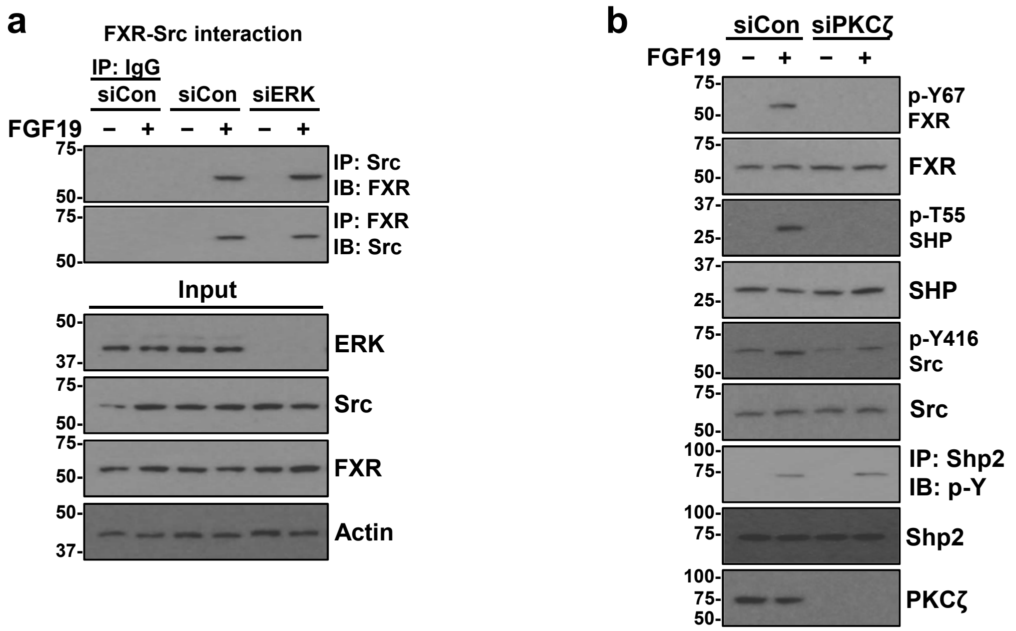

Supplementary Figure 7. FGF19 signaling studies in primary mouse hepatocytes: (a) The interaction of FXR with Src is not affected by downregulation of ERK. PMH from C57BL/6 mice were transfected with siRNA for ERK as indicated, and then the cells were treated with 50 ng ml^{-1} FGF19 for 10 min. FXR and Src in anti-Src and anti-FXR immunoprecipitates, respectively, were detected by IB. **(b) Downregulation of PKCζ results in decreased p-Y67-FXR and p-Y416-Src levels.** PMH from C57BL/6 mice were transfected with siRNA for PKCζ as indicated and the cells were treated with 50 ng ml^{-1} FGF19 for 10 min. Protein levels of PKCζ, p-Y67 FXR, FXR, p-SHP (T55), SHP, p-Src (Y416), Src, p-Y Shp2 and Shp2 were detected by IB.

Supplementary Figure 8

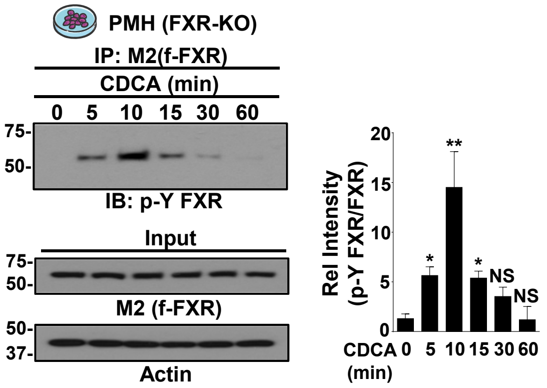

Supplementary Figure 8. Treatment with a primary BA, chenodeoxy cholic acid (CDCA), results in transient Tyr-phosphorylation of FXR in hepatocytes. Ad-flag-FXR was expressed in PMH from FXR-KO mice. Cells were treated with vehicle or 50 µM CDCA for the indicated times, and FXR phosphorylation levels were determined by IP/IB. Relative levels of FXR phosphorylation are shown on the right (mean ± SD, n= 3). Statistical significance was measured using one-way ANOVA with the Bonferroni post-test. *P<0.05, **P<0.01, and NS, statistically not significant.

Supplementary Figure 9

Fig 1. a

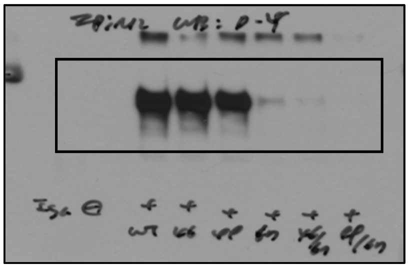

IB: p-Y FXR

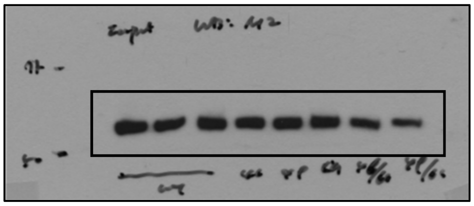

M2 (f-FXR)

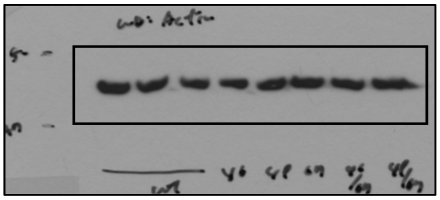

Actin

Fig 1. b

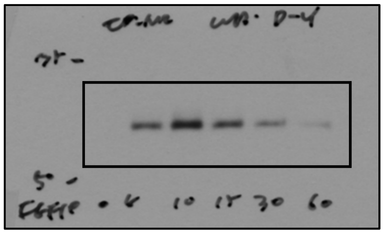

IB: p-Y FXR

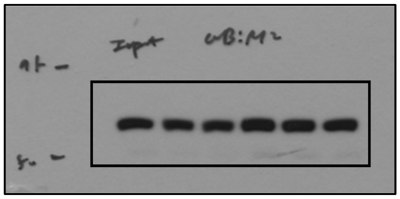

M2 (f-FXR)

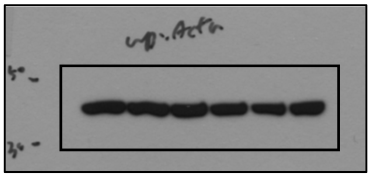

Actin

Fig 1. d

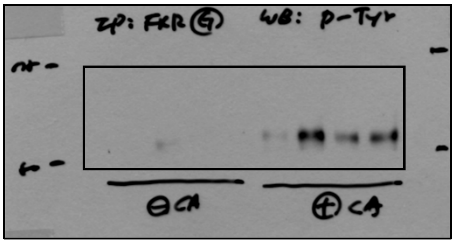

IB: p-Y FXR

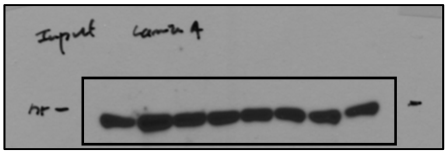

Lamin A

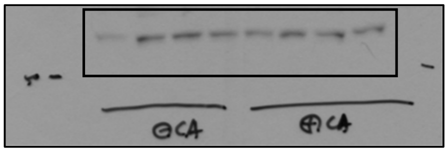

FXR

Supplementary Figure 9. Full size immunoblots of cropped blots in the main manuscript figures. Full size immunoblots of cropped blots for Fig. 1a, 1b, and 1d.

Supplementary Figure 10

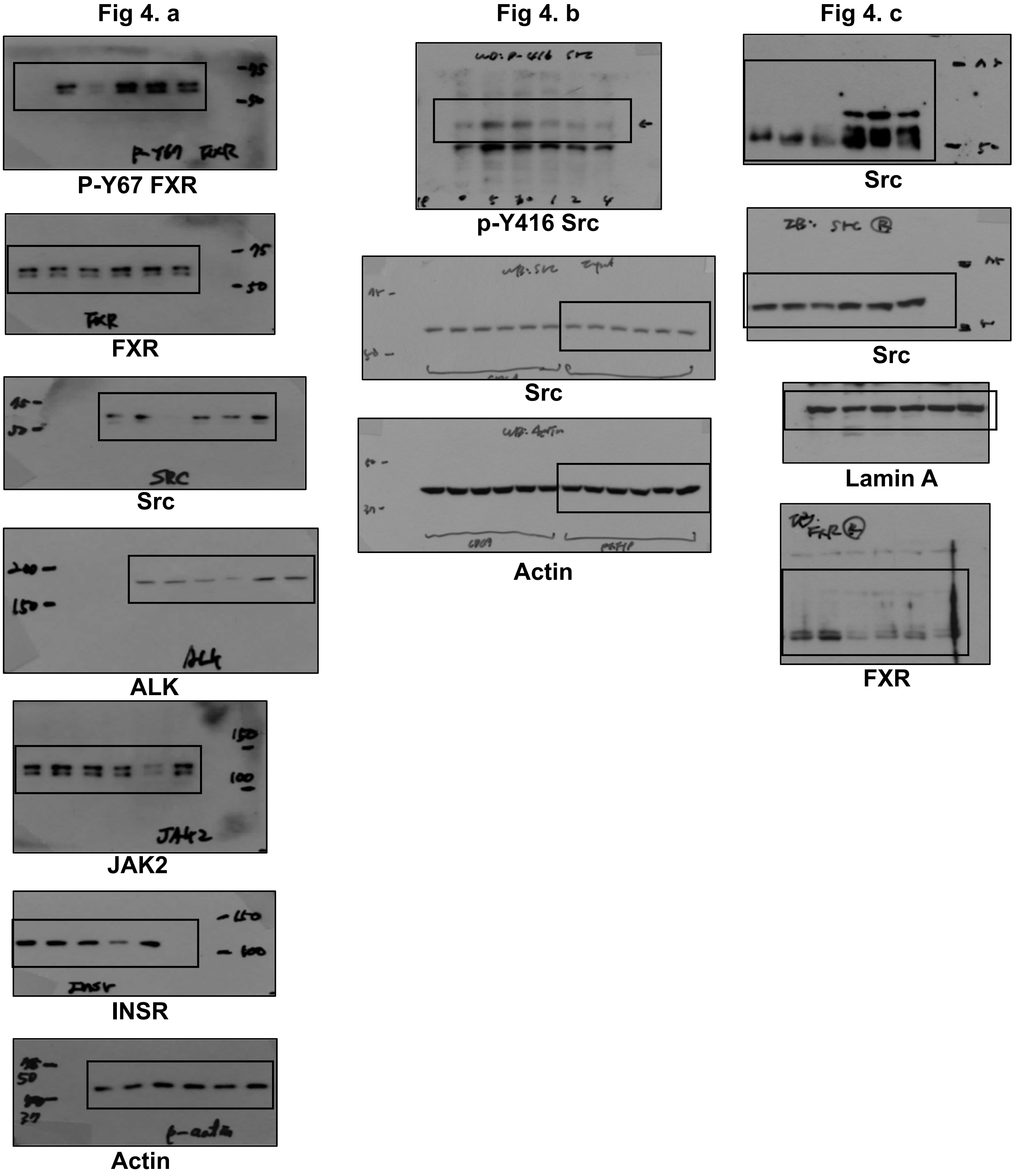

Supplementary Figure 10. Full size immunoblots of cropped blots in the main manuscript figures. Full size immunoblots of cropped blots for Fig. 4a, 4b, and 4c.

Supplementary Figure 11

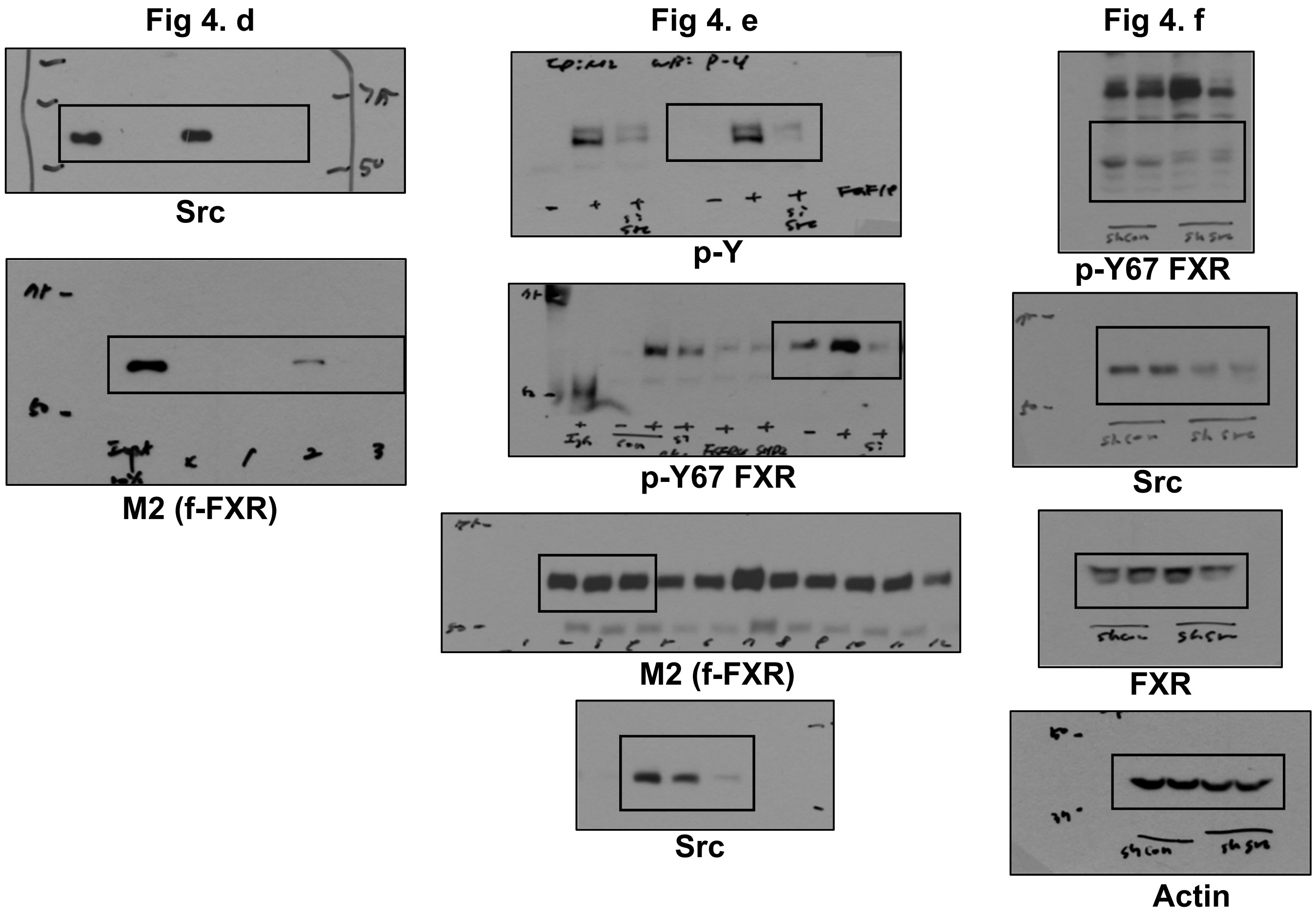

Supplementary Figure 11. Full size immunoblots of cropped blots in the main manuscript figures. Full size immunoblots of cropped blots for Fig. 4d, 4e, and 4f.

Supplementary Figure 12

Fig 4. g

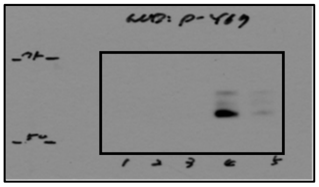

p-Y67 FXR

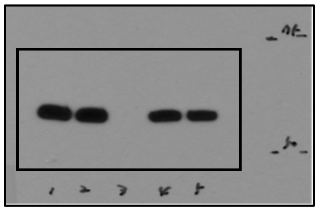

M2 (f-FXR)

Fig 4. j

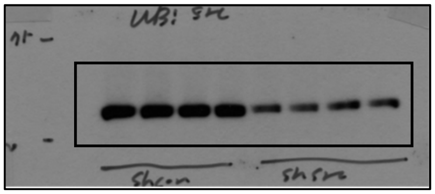

Src

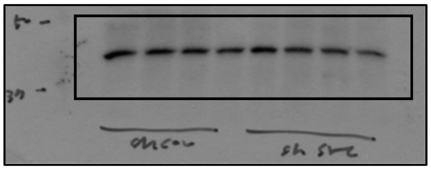

Actin

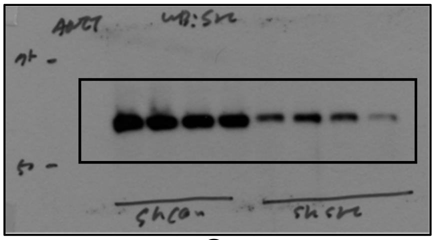

Src

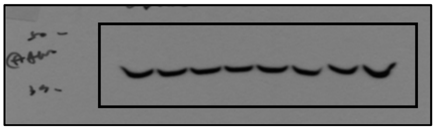

Actin

Supplementary Figure 12. Full size immunoblots of cropped blots in the main manuscript figures. Full size immunoblots of cropped blots for Fig. 4g and 4j.

Supplementary Figure 13

Fig 5. b

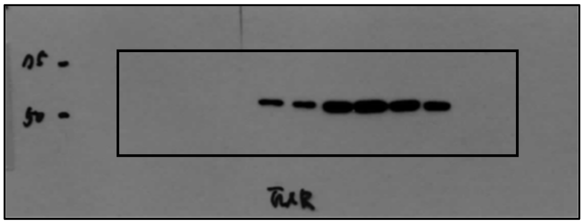

FXR

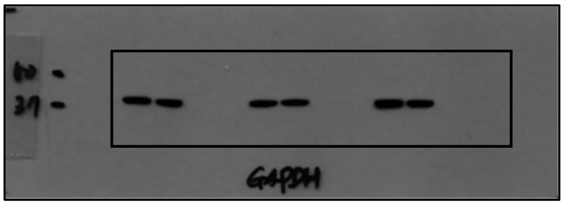

GAPDH

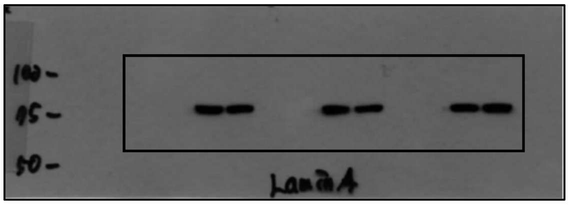

Lamin A

Fig 5. d (left)

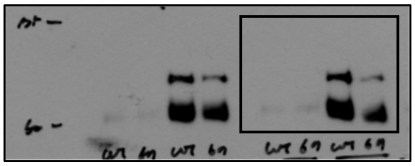

M2 (f-FXR)

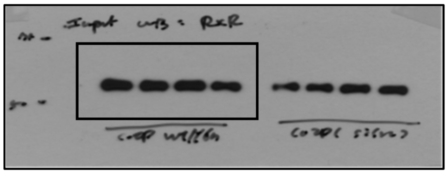

RXRα

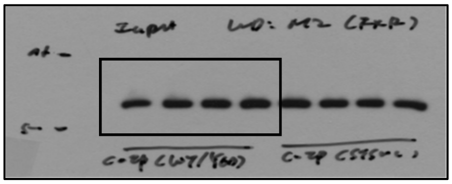

M2 (f-FXR)

Fig 5. d (right)

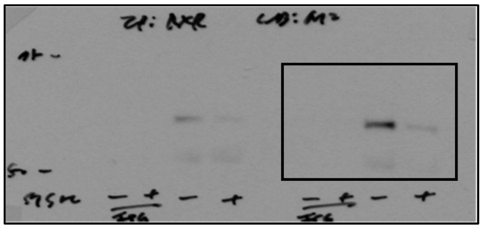

M2 (f-FXR)

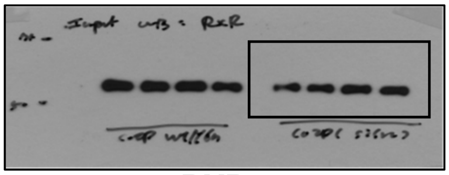

RXRα

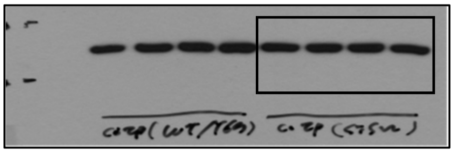

M2 (f-FXR)

Supplementary Figure 13. Full size immunoblots of cropped blots in the main manuscript figures. Full size immunoblots of cropped blots for Fig. 5b and 5d.

Supplementary Figure 14

Fig 6. a

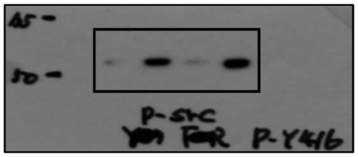

p-Y416 Src

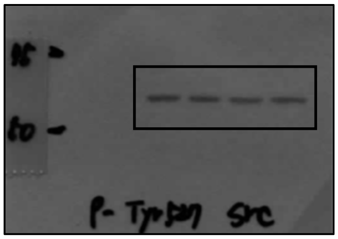

p-Y527 Src

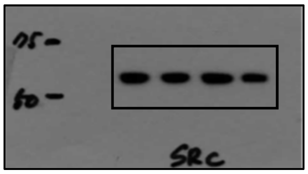

Src

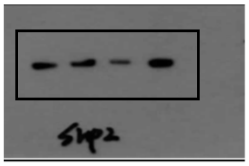

Shp2

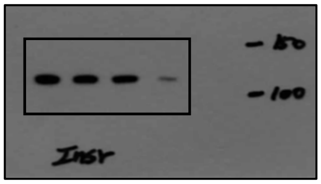

INSR

Fig 6. b

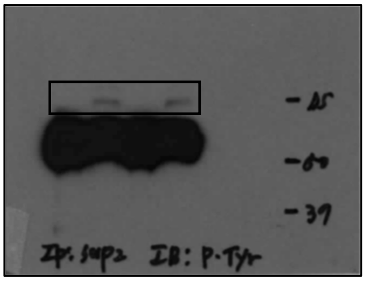

IP: Shp2 IB: p-Y

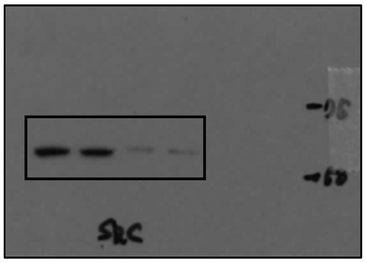

Src

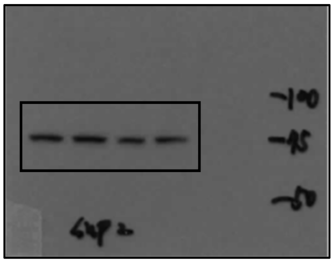

Shp2

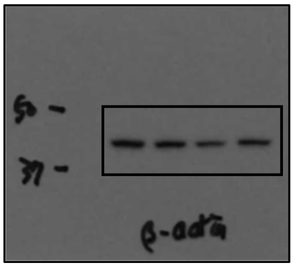

Actin

Supplementary Figure 14. Full size immunoblots of cropped blots in the main manuscript figures. Full size immunoblots of cropped blots for Fig. 6a and 6b.

Supplementary Figure 15

Fig 6. d

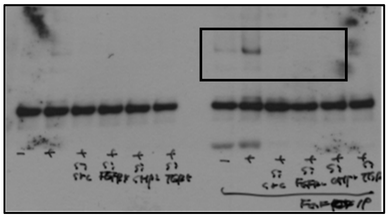

p-Y FXR

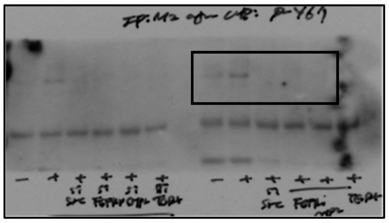

p-Y67 FXR

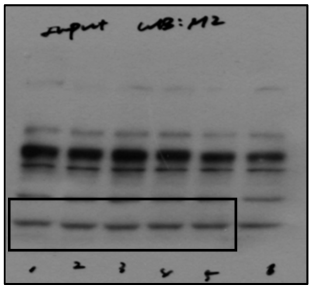

M2 (f-FXR)

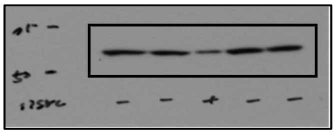

Src

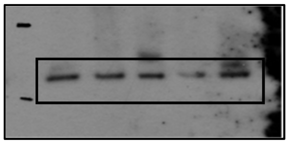

FGFR4

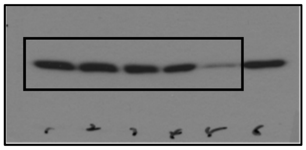

Shp2

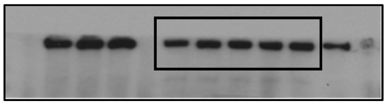

Actin

Fig 6. h

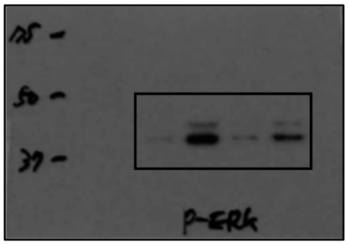

p-ERK

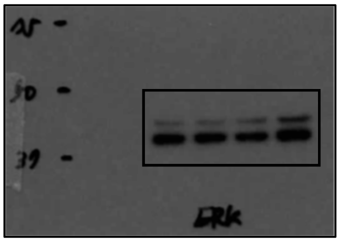

ERK

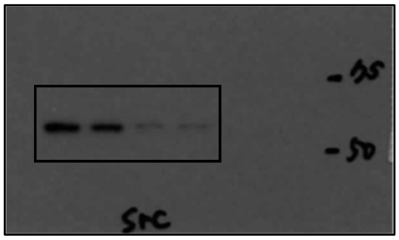

Src

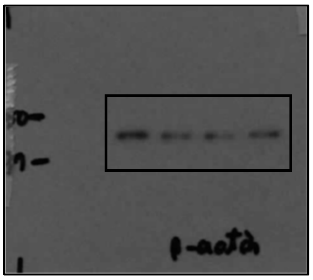

Actin

Supplementary Figure 15. Full size immunoblots of cropped blots in the main manuscript figures. Full size immunoblots of cropped blots for Fig. 6d and 6h.

Supplementary Figure 16

Supplementary Figure 4c

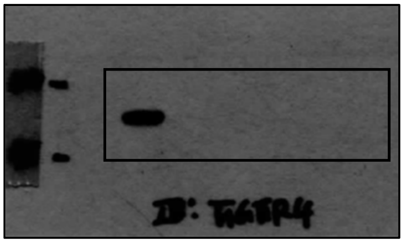

FGFR4

Supplementary Figure 8

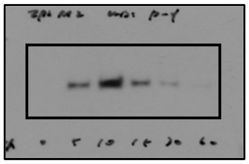

p-Y FXR

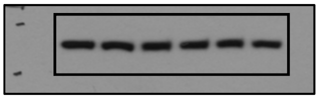

M2 (f-FXR)

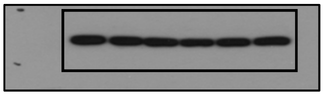

Actin

Supplementary Figure 5a

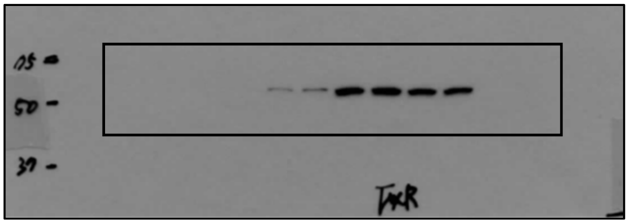

FXR

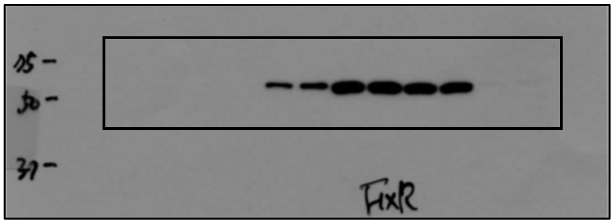

FXR (longer exposure)

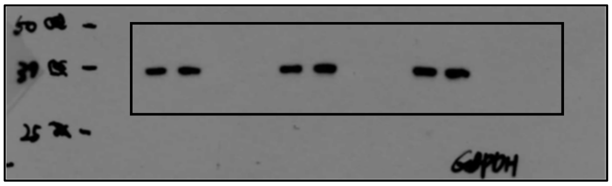

GAPDH

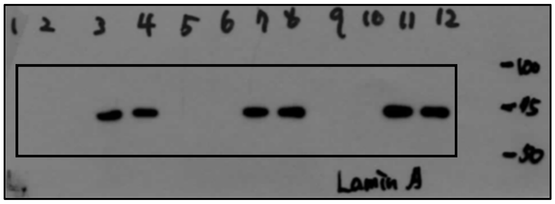

Lamin A

Supplementary Figure 16. Full size immunoblots of cropped blots in the Supplementary figures. Full size immunoblots of cropped blots for Supplementary Figure. 4c, 5a and 8.
